# Discovering multi–level structures in bio-molecular data through the Bernstein inequality

**DOI:** 10.1186/1471-2105-9-S2-S4

**Published:** 2008-03-26

**Authors:** Alberto Bertoni, Giorgio Valentini

**Affiliations:** 1 DSI, Dipartimento di Scienze dell' Informazione, Universitá degli Studi di Milano, Via Comelico 39, Milano, Italy

## Abstract

**Background:**

The unsupervised discovery of structures (i.e. clusterings) underlying data is a central issue in several branches of bioinformatics. Methods based on the concept of stability have been recently proposed to assess the reliability of a clustering procedure and to estimate the “optimal” number of clusters in bio-molecular data. A major problem with stability-based methods is the detection of multi-level structures (e.g. hierarchical functional classes of genes), and the assessment of their statistical significance. In this context, a chi-square based statistical test of hypothesis has been proposed; however, to assure the correctness of this technique some assumptions about the distribution of the data are needed.

**Results:**

To assess the statistical significance and to discover multi-level structures in bio-molecular data, a new method based on Bernstein's inequality is proposed. This approach makes no assumptions about the distribution of the data, thus assuring a reliable application to a large range of bioinformatics problems. Results with synthetic and DNA microarray data show the effectiveness of the proposed method.

**Conclusions:**

The Bernstein test, due to its loose assumptions, is more sensitive than the chi-square test to the detection of multiple structures simultaneously present in the data. Nevertheless it is less selective, that is subject to more false positives, but adding independence assumptions, a more selective variant of the Bernstein inequality-based test is also presented. The proposed methods can be applied to discover multiple structures and to assess their significance in different types of bio-molecular data.

## Background

Unsupervised cluster analysis of bio-molecular data is one of the main and well-established research lines in bioinformatics [1]. Classes of co-expressed genes, classes of functionally related proteins, or subgroups of patients with malignancies differentiated at bio-molecular level can be discovered through clustering algorithms, and several other tasks related to the analysis of bio-molecular data require the development and application of unsupervised clustering techniques [2-4]. Anyway, in most bioinformatics problems, we need to assess the reliability of the discovered clusters, as well as the proper selection of the “natural” number of clusters underlying the data [5].

Recently, several methods based on the concept of stability have been proposed to estimate the “optimal” number of clusters [6,7]: multiple clusterings are obtained by introducing perturbations into the original data, and a clustering is considered reliable if it is approximately maintained across multiple perturbations. Different procedures may be applied to randomly perturb the data, ranging from bootstrapping techniques [8], to noise injection into the data [9] or random projections into lower dimensional subspaces [10,11].

A major problem with stability-based methods is the detection of multi-level structures underlying the data (e.g. hierarchical subclasses of diseases, or hierarchical functional classes of genes). For instance, it is possible that data exhibit a hierarchical structure, with subclusters inside other clusters, and we need to detect these multi-level structures, possibly estimating their reliability and statistical significance. In [7], it is proposed a χ^2^-based statistical test of hypothesis to assess the significance of the “optimal” number of clusters and to discover multiple structures simultaneously present in bio-molecular data; however, by this approach, on one hand some assumptions about the distribution of the similarity measures are needed to estimate the reliability of the obtained clusterings, and on the other hand test results depend on the choice of user-defined parameters.

In this contribution we propose a distribution-free approach that does not assume any “a priori” distribution of the similarity measures, and that does not require any user-defined additional parameter. The proposed approach is based on the classical Bernstein inequality [12], and for its loose assumptions about the distribution of the data may in principle be applied to any unsupervised model order selection problem. More precisely the proposed stability-based method may be applied to several tasks related to the unsupervised analysis of complex bio-molecular data: (a) the assessment of the reliability of a given clustering solution; (b) the clustering model order selection, that is the discovery of the “natural” number of clusters in the data; (c) the assessment of the statistical significance of a given clustering solution; (d) the discovery of multiple structures underlying the data, i.e. the detection of multiple reliable clustering solutions at a given significance level.

## Methods

In the following sections we summarize the characteristics of the stability-based procedures for the assessment of the reliability of clusterings, and we introduce our proposed method based on the Bernstein inequality.

### Model order selection through stability based procedures

Let be *C* a clustering algorithm, ρ(*D*) a given random perturbation procedure applied to a data set *D* and *sim* a suitable similarity measure between two clusterings (e.g. the Jaccard similarity [13]). Among the random perturbations we recall random projections from a high dimensional to a low dimensional subspace [14], or bootstrap procedures to sample a random subset of data from the original data set *D*[8]. Fixing an integer *k* (the number of clusters), we define *S_k_* (0 ≤ *S_k_* ≤ 1) as the random variable given by the similarity between two *k*-clusterings obtained by applying a clustering algorithm *C* to data pairs *D*_1_ and *D*_2_ obtained by randomly and independently perturbing the original data *D*.

If *S_k_* is concentrated close to 1, then the corresponding clustering is stable with respect to a given controlled perturbation and hence it is reliable. This idea, mutuated by a qualitative method proposed in [15], can be formalized using the integral *g*(*k*) of the cumulative distribution *F_k_* of *S_k_*[7]:

$$g\left(k\right)\;=\;\int_{0}^{1}F_{k}\left(s\right)ds\;$$

If *g*(*k*) is close to 0 then the values of the random variable *S_k_* are close to 1 and hence the *k*-clustering is stable, while for larger values of *g*(*k*) the *k*-clustering is less reliable. This observation comes from the following fact:

**Fact**: $E\left[S_{k}\right]\;=\;1\;-\;g\left(k\right),\;\;\;\;\;\;\;\;\;\;\;\;\;Var\left[S_{k}\right]\;\leq \;g\left(k\right)\;\left(1\;-\;g\left(k\right)\right).$

Proof:

Let be *f_k_*(*s*) the probability density function of *S_k_*; then

$$E\left[S_{k}\right]\;=\;\int_{0}^{1}sf_{k}\left(s\right)ds\;\;=\;\int_{0}^{1}sF_{k}^{'}\left(s\right)ds\;\;=\;1\;-\;\int_{0}^{1}F_{k}\left(s\right)ds\;\;=\;1\;-\;g\left(k\right)$$

Moreover:

$$Var\left[S_{k}\right]\;=\;E\left[S_{k}^{2}\right]\;-\;E{\left[S_{k}\right]}^{2}\;\leq \;E\left[S_{k}\right]\;-\;E{\left[S_{k}\right]}^{2}\;=\;g\left(k\right)\;\left(1-g\left(k\right)\right)$$

☐.

Hence, *g*(*k*) ≃ 0 implies *Var*[*S_k_*] ≃ 0. As a consequence, *g*(*k*) or equivalently *E*[*S_k_*] can be used as a good index of the reliability of the *k*-clusterings (clusterings with *k* clusters). *E*[*S_k_*] may be estimated by the empirical mean ξ*_k_* of *n* replicated similarity measures between pairs of perturbed clusterings:

$$\xi_{k}\;=\;\sum_{j=1}^{n}\frac{S_{kj}}{n}$$

where *S_kj_* represents the similarity between two *k*-clusterings obtained through the application of the algorithm *C* to a pair of perturbed data.

We may perform a sorting of the ξ*_k_*:

$$\left(\xi_{2},\;\xi_{3,\;...\;,\;}\xi_{H+1}\right)\;\;\underset{\to }{sort}\;\left(\xi_{p\left(1\right)},\;\xi_{p\left(2\right)}\;,...\;,\;\;\xi_{p\left(H\right)}\right)$$

where *p* is an index permutation such that ξ_*p*(1)_ ≥ ξ_*p*(2)_ ≥ … ≥ ξ_*p*(*H*)_. In this way we obtain an ordering of the clusterings, from the most to the least reliable one.

Exploiting this ordering, we proposed a χ^2^-based statistical test to detect and to estimate the statistical significance of multiple-structures discovered by clustering algorithms [7]. The main drawbacks of this approach consists in an implicit normality assumption for the distribution of the *S_k_* (random variables that measure the similarity between two perturbed *k*-clusterings, see above), and in a user defined threshold parameter that determines when two *k*-clusterings can be considered similar and “stable”. Indeed, in general we have no guarantee that the *S_k_* random variables are normally distributed; moreover the “optimal” choice of the threshold parameter seems to be application dependent and may affect the overall test results.

In this contribution, to address these problems we propose a new statistical method that, adopting a stability-based approach, makes no assumptions about the distribution of the random variables and does not require any user-defined threshold parameter.

### Hypothesis testing based on Bernstein inequality

We briefly recall the Bernstein inequality, because this inequality is used to build-up our proposed hypothesis testing procedure.

**Bernstein inequality**. If *Y*_1_, *Y*_2_, …, *Y_n_* are independent random variables s.t. 0 ≤ *Y_i_* ≤ 1, with $\mu \;=\;E\left[Y_{i}\right],\;\sigma^{2}\;=\;Var\left[Y_{i}\right],\;\bar{Y}\;=\;\sum Y_{i}/n$ then

$$Prob\left\{\bar{Y}\;-\;\mu \;\geq \;\Delta \right\}\;\leq e{\;}^{\frac{-n\Delta^{2}}{2\sigma^{2}+\;2/3\;\Delta }}$$

Using the Bernstein inequality, we would estimate if for a given *r*, 2 ≤ *r* ≤ *H*, there exists a statistically significant difference between the reliability of the best *p*(1) clustering and the *p*(*r*) clustering (eq. 3). In other words we may state the null hypothesis *H*_0_ and the alternative hypothesis in the following way:

*H*_0_: *p*(1) clustering is not more reliable than *p*(*r*) clustering, that is *E*[*S*_*p*(1)_] ≤ *E*[*S*_*p*(*r*)_]

*H_a_*: *p*(1) clustering is more reliable than *p*(*r*) clustering, that is *E*[*S*_*p*(1)_] >*E*[*S*_*p*(*r*)_]

To this end, consider the following random variables:

$$P_{i}\;=\;S_{p\left(1\right)}\;-\;S_{p\left(i\right)}\;\;\;\text{and}\;\;\;X_{i}\;=\;\xi_{p\left(1\right)}\;-\;\xi_{p\left(i\right)}$$

We start considering the first and last ranked clustering *p*(1) and *p*(*H*). In this case the above null hypothesis *H*_0_ becomes: *E*[*S*_*p*(1)_] ≤ *E*[*S*_*p*(*H*)_], or equivalently *E*[*S*_*p*(1)_] − *E*[*S*_*p*(*H*)_] = *E*[*P_H_*] ≤ 0. The distribution of the random variable *X_H_* (eq. 5) is in general unknown; anyway note that in the Bernstein inequality no assumption is made about the distribution of the random variables *Y_i_* (eq. 4). Hence, fixing a parameter Δ ≥ 0, considering true the null hypothesis *E*[*P_H_*] ≤ 0, and using Bernstein inequality, we have:

$$Prob\left\{X_{H}\geq \;\Delta \right\}\;\;\leq \;Prob\;\left\{X_{H}\;-\;E\left[P_{H}\right]\geq \;\Delta \right\}\;\;\;\leq \;e^{\frac{-n\;\Delta^{2}}{2\sigma^{2}+2/3\Delta }}$$

Considering an instance (a measured value) ${\hat{X}}_{H}$ of the random variable *X_H_*, if we let $\Delta ={\hat{X}}_{H}$ we obtain the following probability of type I error:

$$P_{err}\left\{X_{H}\;\geq \;{\hat{X}}_{H}\right\}\;\leq \;e^{\frac{-\;n\;{\hat{X}}_{H}^{2}}{\;2\sigma_{H}^{2}+\;2/3\;{\hat{X}}_{H}}}$$

with $\sigma_{H}^{2}\;=\;\sigma_{p\left(1\right)}^{2}\;+\;\sigma_{p\left(H\right)}^{2}.$

If $P_{err}\left\{X_{H}\;\geq \;{\hat{X}}_{H}\right\}<\;\alpha ,$ we reject the null hypothesis: a significant difference between the two clusterings is detected at α significance level and we continue by testing the *p*(*H* − 1) clustering. More in general if the null hypothesis has been rejected for the *p*(*H* − *r* + 1) clustering, 1 ≤ *r* ≤ *H* − 2 then we consider the *p*(*H* − *r*) clustering, and using the Boole inequality, we can estimate the type I error:

$$P_{err}\left(H\;-\;r\right)\;=\;Prob\left\{\underset{H-r\leq i\leq H}{\vee }\;X_{i}\;\geq \;{\hat{X}}_{i}\right\}\;\leq \;\sum_{i=H-r}^{H}Prob\left\{X_{i}\;\geq \;{\hat{X}}_{i}\right\}\;\leq \sum_{i=H-r}^{H}e^{\frac{-\;n\;{\hat{X}}_{i}^{2}}{2\sigma_{i}^{2}+\;2/3\;{\hat{X}}_{i}\;}}$$

As in the previous case, if *P_err_*(*H* − *r*) < α we reject the null hypothesis: a significant difference is detected between the reliability of the *p*(1) and *p*(*H* − *r*) clustering and we iteratively continue the procedure estimating *P_err_*(*H* − *r* − 1).

This procedure stops if either of these cases succeeds:

I) The null hypothesis is rejected till *r* = *H* − 2, that is ∀*r*, 1 ≤ *r* ≤ *H* − 2, *P_err_*(*H* − *r*) < α: all the possible null hypotheses have been rejected and the only reliable clustering at α-significance level is the top ranked one, that is the *p*(1) clustering.

II) The null hypothesis cannot be rejected for *r* ≤ *H* − 2, that is, ∃*r*, 1 ≤ *r* ≤ *H* − 2, *P_err_*(*H* − *r*) ≥ α: in this case the clusterings that are significantly less reliable than the top ranked *p*(1) clustering are the *p*(*r* + 1), *p*(*r* + 2),…, *p*(*H*) clusterings.

Note that in this second case we cannot state that there is no significant difference between the first *r* top-ranked clusterings, since the upper bound provided by the Bernstein inequality is not guaranteed to be tight. To answer to this question, we may apply the χ^2^-based hypothesis testing proposed in [7] to the remaining top ranked clusterings to establish which of them are significant at α level, but in this case we need to assume that the similarity measures between pairs of clusterings are distributed according to a normal distribution.

If we assume that the *X_i_* random variables (eq. 5) are (at least approximately) independent, we can obtain a variant of the previous Bernstein inequality-based approach, that we name *Bernstein ind*. for brevity. By this approach we should in principle obtain lower *p values*, thus assuring lower false positive rates than the *Bernstein* test without independence assumptions.

With these independence assumptions the null hypothesis *H_0_* and the alternative hypothesis for the *Bernstein ind*. test can be formulated as follows:

*H*_0_: ∃*i*, 2 ≤ *i* ≤ *r* ≤ *H* such that *E*[*S*_*p*(1)_] ≤ *E*[*S*_*p*(*r*)_]: it does exist at least one *p*(*i*)-clustering equally or more reliable than the first one in the group of the first *r* ordered clusterings.

*H_a_:* ∀*i*, 2 ≤ *i* ≤ *r* ≤ *H, E*[*S*_*p*(1)_] >*E*[*S*_*p*(*r*)_]: all the clusterings in the group of the first *r* ordered clusterings are less reliable than the first one.

If we assume that the null hypothesis is true, using the independence among the *X_i_* random variables, we may obtain the type I error:

$$P_{err}\left(r\right)\;=\;Prob\left\{\underset{2\leq i\leq r}{\Lambda }X_{i}\;\geq \;{\hat{X}}_{i}\right\}\;=\;\prod_{i=2}^{r}Prob\left\{X_{i}\;\geq \;{\hat{X}}_{i}\right\}\;\leq \;\prod_{i=2}^{r}\;e^{\frac{-n{\hat{X}}_{i}^{2}}{2\sigma_{i}^{2}+\;2/3{\hat{X}}_{i}}}$$

Starting from *r* = *H*, if *P_err_(r) < α* we reject the null hypothesis: a significant difference is detected between the reliability of the *p*(1) and the other first *r*-clustering and we iteratively continue the procedure estimating *P_err_*(*r* − 1). As in the *Bernstein* test, the procedure is iterated until we remain with a single clustering (and this will be the only significant one), or until *P_err_*(*r*) ≥ α and in this case we cannot reject the null hypothesis and the first *r* clusterings can be considered equally reliable. Note that, strictly speaking, in this case we can only say that at least one of the first *r* clusterings is equally or more reliable than the first one.

## Results and discussion

In this section we apply the *Bernstein* test to synthetic and DNA microarray data analysis, and compare it to the previously proposed χ^2^-based test [7]. For the experiments we used the *mosclust* R package [16], and all the data used in the experiments are available from the authors.

### Analysis of hierarchical structures in synthetic data

**Figure 1:**
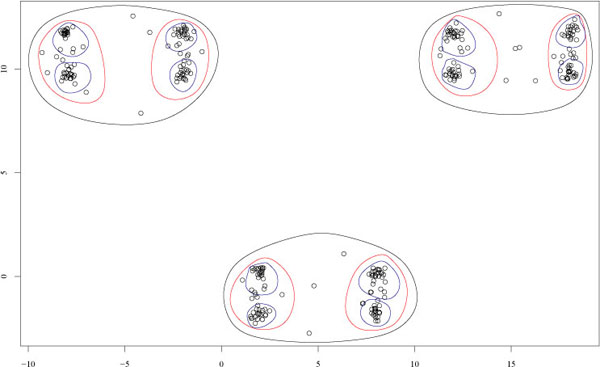
Synthetic data set: a three-level hierarchical structure with 3, 6 and 12 clusters.

In order to show the effectiveness of the proposed approach we consider synthetic data with a priori known multi-level hierarchical structure. To this end we generated a two-dimensional synthetic data set with a three-level hierarchical structure (Fig. 1) using the *clusterv* R package [17]: at a first level three large clusters are present in the data (black ovals); at a second level we have a 6-clustering (red ovals) and finally a third-level 12-clustering may be detected (blue ovals).

**Figure 2:**
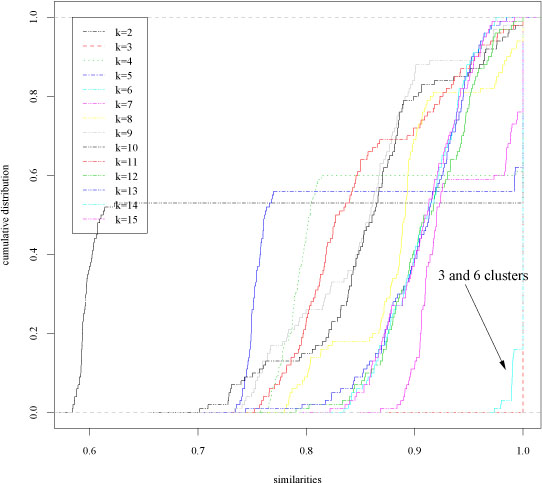
Synthetic data set: empirical cumulative distribution functions of the similarity measures for different number of clusters (Hierarchical clustering).

We performed analysis of the stability of the clusters (see Section *Model order selection through stability based procedures*), by applying random subsample techniques to perturb the data (100 subsample pairs each composed by 80 % of the data randomly drawn without replacement) and Ward's hierarchical clustering algorithm [18] with dendrogram cuts from *k* = 2 to *k* = 15 clusters. Then we computed the similarity indices for each *k* from 2 to 15: their empirical cumulative distribution is shown in Fig. 2. From Fig. 2 we may observe that 3 and 6-clusterings similarities are closely concentrated near 1, thus showing that these clusterings are clearly detectable by the hierarchical clustering algorithm. Indeed both χ^2^-based and *Bernstein*-based test of hypothesis select these clusterings at 10^−5^ significance level. Nevertheless, the *Bernstein* test selects also the 7-clustering (false positive) and the 12-clustering (true positive) (Table 1). As a second experiment we considered a 1000-dimensional synthetic multivariate gaussian distributed data set. These data are characterized by a two-level hierarchical structure: at a first level we have two main clusters with inside each one three other clusters, thus resulting in a second level 6-clustering. Each of the six second-level clusters is distributed according to a hyperspherical gaussian distribution and each cluster contains only 20 examples, thus resulting in a sparse data set (low number of examples in a high dimensional space). We applied the Prediction Around Medoids clustering algorithm [19], and we perturbed the data through *Bernoulli* random projections [7], from a 1000 to a 479-dimensional subspace, considering the reliability of clusterings composed from 2 to 10 clusters. In this case both the χ^2^-based and the *Bernstein* based iterative procedures correctly detect 2 and 6-clusterings at 10 −4 significance level. With these high dimensional data the Bernstein test is not subject to false positives, but also the *χ^2^* test correctly detects all the structures underlying the data.

**Table 1:** Synthetic data: comparison of the χ^2^ and Bernstein inequality-based tests. * Bernstein ind*. stands for the *Bernstein* test with assumption of independence between the random variables representing the empirical mean of the similarity measures.

**Test**	**Structures discovered** (10^−5^ significance level)
χ^2^	3-clustering
	6-clustering
*Bernstein ind*.	3-clustering
	6-clustering
	7-clustering
*Bernstein*	3-clustering
	6-clustering
	7-clustering
	12-clustering

### Discovery of multi-level structures in DNA microarray data

As an example of the application of the Bernstein test to the discovery of multiple structures in bio-molecular data, we consider two classical DNA microarray data sets: *Leukemia*[20] and *Lymphoma*[21]. The *Leukemia* data set is composed by a group of 25 acute myeloid leukemia (AML) samples and another group of 47 acute lymphoblastic leukemia (ALL) samples, that can be subdivided into 38 B-Cell and 9 T-Cell subgroups, resulting in a two-level hierarchical structure.

We applied both resampling and random projections to lower dimensional subspaces to perturb the original data using the *R* package *mosclust *[16] that implements the Bernstein-based test and the stability measures described in Sect. *Model order selection through stability based procedures*.

**Figure 3:**
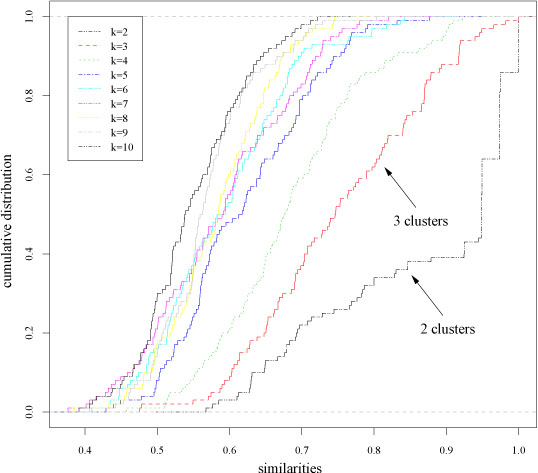
Leukemia data set. Empirical cumulative distributions of the similarity measures for different numbers of clusters *k*.

Fig. 3 shows the empirical cumulative distributions of the similarity values and Table 2 the p values of the clusterings sorted according to their ξ values (eq. 2), using *Bernoulli* random projections [7]. Our proposed procedure detects the 2 – *clustering* as the most reliable at 0.01 significance level, according to the fact that two biologically meaningful groups (ALL, acute lymphoblastic leukemia and AML, acute myeloid leukemia) are present in the data. Choosing a significance level α = 10^−5^ we cannot reject the null hypothesis that a 2-clustering is less or equally reliable than a 3-clustering: in this case 2 structures (2 and 3-clusterings) are detected as simultaneously present in the data, reflecting the biological fact that ALL can be subdivided into two subclasses (B-cell and T-cell ALL).

**Table 2:** Leukemia data set: empirical means (ξ) and p values computed according to the Bernstein inequality.

**Num.clusters**	**p values**	ξ
2	–––-	0.8664
3	1.0561e-04	0.7521
4	1.2165e-08	0.6850
5	1.0554e-12	0.6196
6	3.9321e-14	0.5922
7	1.7630e-14	0.5878
8	2.3732e-15	0.5822
9	2.7570e-16	0.5690
10	1.6297e-17	0.5491

**Figure 4:**
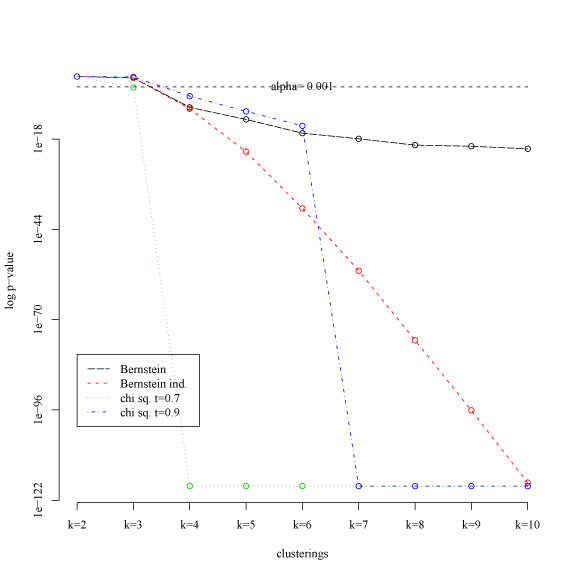
K-means clustering: log p value computed for χ^2^-based and *Bernstein*-based statistical tests. Ordinate: log p value; abscissa: number of clusters sorted according the computed similarity means.

**Figure 5:**
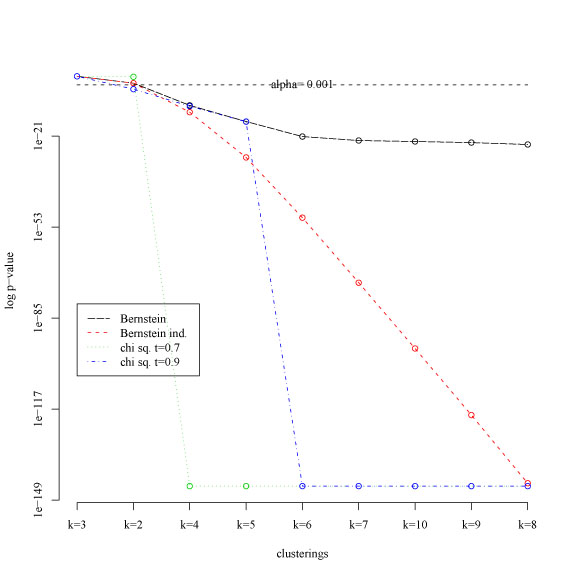
PAM clustering: log p value computed for χ^2^-based and *Bernstein*-based statistical tests. Ordinate: log p value; abscissa: number of clusters sorted according the computed similarity means.

The results obtained with two variants of the χ^2^[7] and *Bernstein* based statistical tests are compared in Fig. 4 (k-means algorithm) and Fig. 5 (PAM, Prediction Around Medoids algorithm [19]) : log p values are represented in ordinate, while in abscissa the number of clusters are sorted according to the empirical mean of the corresponding pairwise similarities (eq. 2). In both figures a straight horizontal dashed line represents a significance level α = 0.001: k-clusterings above the dashed line are significant, that is their reliability significantly differ from the k-clusterings below the dashed horizontal line. Note that the k-means (Fig. 4) and PAM (Fig. 5) clustering algorithms provide a slightly different ranking of the similarity indices, but in most cases 2 and 3 clusterings are considered significantly more reliable than the others, according to the biological characteristics of the data. The Bernstein test, due to its more general assumptions (no particular distributions and no independence are assumed for the random variables that represent the similarity between clusterings) is less selective (in the sense that it may consider reliable a larger number of k-clusterings) than the χ^2^-based test that make assumptions about the distribution of the random variables. This is confirmed by the fact that *Bernstein* p values decrease more slowly with respect to the χ^2^ test (Fig. 4 and 5), thus resulting in a better sensitivity to multiple structures present in the data. The main drawback of this behaviour is the larger probability of false positives. Note that the *Bernstein ind*. test shows an intermediate trend between the *Bernstein* and χ^2^ test (red lines in Fig. 4 and 5): the assumption of independence between the random variables yields a more selective Bernstein inequality-based test less subject to false positives, but potentially less sensitive to multiple structures underlying the data.

**Figure 6:**
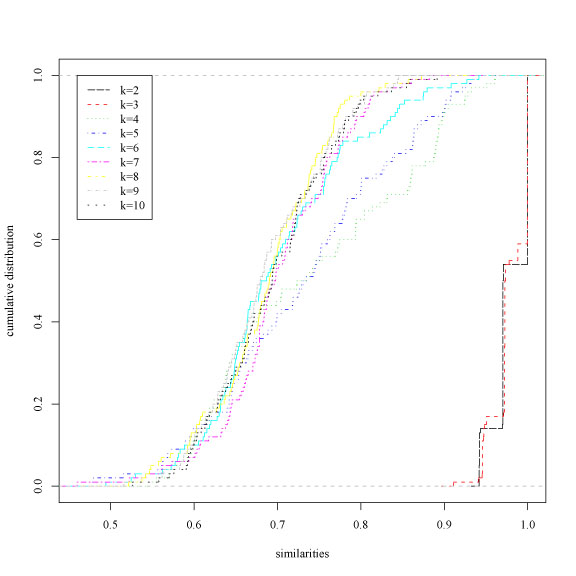
Lymphoma data set. Empirical cumulative distribution functions of the similarity measures for different number of clusters *k*. Perturbation technique: random projections

**Figure 7:**
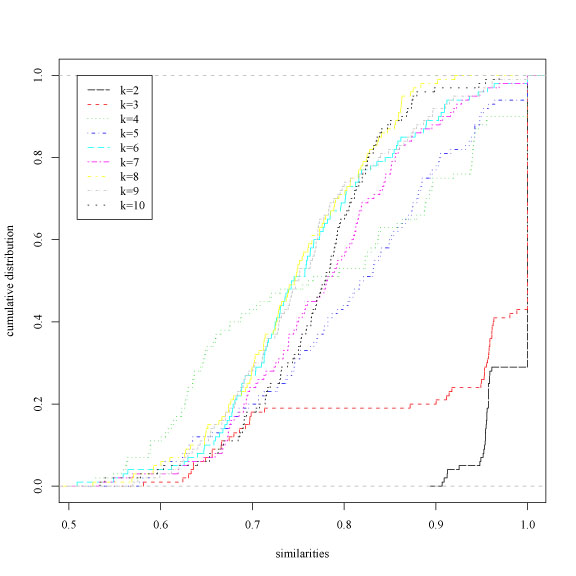
Lymphoma data set. Empirical cumulative distribution functions of the similarity measures for different number of clusters *k*. Perturbation technique: resampling

The *Lymphoma* gene expression data set [21] comprises three different lymphoid malignancies: Diffuse Large B-Cell Lymphoma (DLBCL), Follicular Lymphoma (FL) and Chronic Lymphocytic Leukemia (CLL). The data provides expression levels for 4026 genes [22], with 62 samples subdivided in 42 DLBCL, 11 CLL and 9 FL. We performed pre-processing of the data according to [21], replacing missing values with 0 and then normalizing the data to zero mean and unit variance across genes. We considered both resampling techniques and random projections to perturb the data. In particular, data have been resampled by randomly drawing 80% of the available data without replacement, and data have been projected using *Bernoulli* projections with ε = 0.2 corresponding to 413-dimensional subspaces. Fig. 6 and 7 show the empirical cumulative distribution of the similarity measures for different numbers of clusters, using the hierarchical Ward's clustering algorithm and respectively *Bernoulli* random projections (Fig. 6) and subsampling perturbation techniques (Fig. 7). Considering random projections both the *Bernstein* and χ^2^-based tests correctly select 2 and 3-clusterings at 0.001 significance level. On the contrary, using subsampling techniques only the *Bernstein* inequality based test select as significant both 2 and 3-clusterings, while the χ*^2^* tests select only the 2-clustering (Table 3). These results confirm that the *Bernstein* test is more sensitive to multiple structures underlying the data.

**Table 3:** Lymphoma data: comparison of the χ^2^ and Bernstein inequality-based tests. *t* represents the threshold level for the χ^2^-based test.

**Test**	**Structures discovered** (0:001 significance level)

χ^2^ (t=0.9)	2-clustering
χ^2^ (t=0.7)	2-clustering
*Bernstein ind*.	2-clustering
	3-clustering
*Bernstein*	2-clustering
	3-clustering

Considering the *Leukemia* and *Lymphoma* data sets, the proposed *Bernstein* test achieves results competitive with state-of-the-art stability methods proposed in the literature. Indeed the *Model Explorer* algorithm, based on subsampling techniques, correctly detect only the 2-clustering structure both in *Leukemia* and *Lymphoma*[15]. Another subsampling-based method (*Figure of Merit*[23]) detects 2, 8 and 19-clusterings in *Leukemia* and 2 and 9-clusterings in *Lymphoma*. Stability methods that apply supervised algorithms to assess the quality of the discovered clusterings correctly detect only a 3-clustering in *Leukemia* and a 2-clustering in *Lymphoma*[6,24]. Our previously proposed χ^2^-based test correctly detects both 2 and 3-clusterings in both data sets, if random projections are used as perturbation method, but it fails to detect the 3-clustering in *Lymphoma* when subsampling techniques are applied. On the contrary, the *Bernstein* test discovers both the two-level structures in *Leukemia* and *Lymphoma*, independently of the applied perturbation method.

The experimental results with both synthetic and gene expression data support the hypothesis that the *Bernstein* test is more sensitive to multiple structures underlying the data. Indeed in the first experiment with synthetic data it correctly predicts also the third level of structure, that is the 12-clustering; on the other hand it is subject to false positives, as shown by the wrong discovery of a 7-clustering (Table 1). These results are confirmed by the fact that *Bernstein* p values decrease more slowly with respect to the χ^2^ test (Fig. 4 and 5): in this way for a given significance level it is likely that the *Bernstein* test selects larger sets of structures underlying the data. The risk of an increased rate of false positives may be balanced by the assumption of independence between the random variables, yielding to the proposed *Bernstein ind*. test (eq. 8), less subject to false positives, but potentially less sensitive to multiple structures underlying the data.

In real applications to complex bio-molecular data, we suggest to apply both *Bernstein*-based and χ^2^-based procedures: structures discovered by both tests are likely to be significant, and *Bernstein*-based tests can discover potential structures not detectable with the more selective χ^2^-based test. Moreover the computational burden due to the application of the χ^2^ and *Bernstein*-based iterative procedures is irrelevant with respect to the execution of clustering algorithms.

## Conclusions

We proposed a test of hypothesis based on Bernstein inequality to estimate if there is a significant difference between the reliability of different clusterings performed on the same data. Our proposed method can be applied to discover multiple or hierarchical structures, using different clustering algorithms and different perturbation methods. Even if in our experiments we applied the *Bernstein* test to the analysis of gene expression data, this approach may be in principle applied to discover multiple structures in any type of complex bio-molecular data. Indeed no user-defined parameters are required, and very loose assumptions are made about the distribution of the data and the distribution of the similarity values used to estimate the stability of the discovered clusterings, thus assuring a reliable application of the method to a large range of bioinformatics problems.

Our experiments with synthetic and gene expression data show that *Bernstein*-based tests are more sensitive than χ^2^-based tests to multiple structures embedded in the data: in this way not self-evident structures may be detected too, as well as subtle relationships between the data. A drawback of the *Bernstein* test is its larger expected rate of false positives, but assuming independence between the empirical means of the similarity values a new test (*Bernstein ind*.), less subject to false positives, has been proposed.

Developments of this work could consist in the adaptation and application of the proposed methods to large scale bioinformatics problems, to discover multiple structures underlying the data when a very large number of clusters is potentially involved.

## Competing interests

The authors declare that they have no competing interests.

## Authors' contributions

The authors equally contributed to this paper.
